# Bves Modulates Tight Junction Associated Signaling

**DOI:** 10.1371/journal.pone.0014563

**Published:** 2011-01-20

**Authors:** Patricia K. Russ, Christopher J. Pino, Christopher S. Williams, David M. Bader, Frederick R. Haselton, Min S. Chang

**Affiliations:** 1 Department of Biomedical Engineering, Vanderbilt University, Nashville, Tennessee, United States of America; 2 Department of Ophthalmology and Visual Sciences, Vanderbilt University, Nashville, Tennessee, United States of America; 3 Department of Cancer Biology, Vanderbilt University, Nashville, Tennessee, United States of America; 4 Department of Cardiovascular Medicine, Vanderbilt University, Nashville, Tennessee, United States of America; University of Illinois at Chicago, United States of America

## Abstract

Blood vessel epicardial substance (Bves) is a transmembrane adhesion protein that regulates tight junction (TJ) formation in a variety of epithelia. The role of TJs within epithelium extends beyond the mechanical properties. They have been shown to play a direct role in regulation of RhoA and ZONAB/DbpA, a y-box transcription factor. We hypothesize that Bves can modulate RhoA activation and ZONAB/DbpA activity through its regulatory effect on TJ formation. Immortalized human corneal epithelial (HCE) cells were stably transfected with Flag-tagged full length chicken Bves (w-Bves) or C-terminus truncated Bves (t-Bves). We found that stably transfected w-Bves and t-Bves were interacting with endogenous human Bves. However, interaction with t-Bves appeared to disrupt cell membrane localization of endogenous Bves and interaction with ZO-1. w-Bves cells exhibited increased TJ function reflected by increased trans-epithelial electrical resistance, while t-Bves cells lost TJ protein immunolocalization at cell-cell contacts and exhibited decreased trans-epithelial electrical resistance. In parental HCE and w-Bves cells ZONAB/DbpA and GEF-H1 were seen at cell borders in the same pattern as ZO-1. However, expression of t-Bves led to decreased membrane localization of both ZONAB/DbpA and GEF-H1. t-Bves cells had increased RhoA activity, as indicated by a significant 30% increase in FRET activity compared to parental HCE cells. ZONAB/DbpA transcriptional activity, assessed using a luciferase reporter probe, was increased in t-Bves cells. These studies demonstrate that Bves expression and localization can regulate RhoA and ZONAB/DbpA activity.

## Introduction

**Figure 1:**
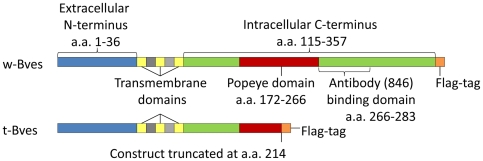
Bves protein constructs. The w-Bves construct contains the full-length chicken Bves protein with a C-terminal Flag tag. The t-Bves construct is truncated within the conserved Popeye domain. Subsequently, the binding domain of the anti-Bves 846 antibody has also been deleted.[3], [4], [9].

Blood vessel epicardial substance (Bves) is a transmembrane adhesion protein that regulates tight junction formation in a variety of epithelia [1], [2]. Bves is classified as an adhesion molecule due to its ability to confer adhesive properties to non-adherent cells [3]. However, analysis of Bves' primary structure does not reveal recognizable motifs or domains that could classify Bves into any known family of adhesion proteins [4], [5]. Bves is now placed into a gene family called the *Popeye Domain Containing* (*Popdc*) family [6]. Family members include *Popdc 1*, *2*, *and 3*, with Bves being the prototypic member and synonymous with *Popdc1* [4]. The Popeye domain, common to all Popdc members, is highly conserved, exhibiting greater than 85% homology among family members [4], [7]. Bves is a 3-span transmembrane protein, and topographic studies by Knight et al verified that the Popeye domain of Bves (aa 172–266), along with a large portion of the carboxyl terminus (aa 115–357), is contained within the cytoplasm (Figure 1) [8]. While relatively little is known regarding the specific role of the Popeye domain, evidence suggests that the intracellular carboxyl terminus is important in subcellular trafficking of Bves to the cell membrane. Bves proteins are capable of homotypic interactions [8], [9], and disruption of Bves-Bves interactions leads to disruption of Bves localization to the cell membrane [9].

Bves appears to regulate cell adhesion through modulation of tight junction (TJ) formation. By altering levels of Bves expression, we observed formation of TJs to be dependent on the levels of Bves in human corneal epithelial cells and human trabecular meshwork cells [9], [10]. Increased Bves expression led to increased TJ protein expression and function, while knock-down of Bves led to decreased TJ function [1], [10].

**Figure 2:**
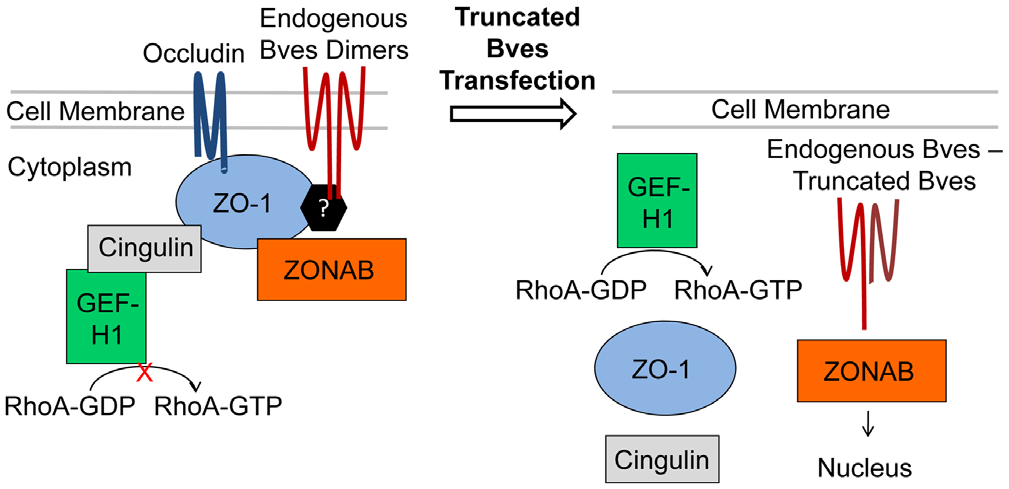
Proposed tight junction signaling pathways modulated by Bves. Bves is a transmembrane adhesion molecule capable of complexing with ZO-1 through an unknown mechanism and modulating tight junction (TJ) formation. We hypothesize that Bves is capable of modulating TJ associated signaling through the regulation of TJ formation (left). TJs are signaling complexes that regulate RhoA activation by sequestering the small GTPase GEF-H1 within the TJ. ZONAB/Dbpa, a Y-box transcription factor, is also sequestered at the TJ by an interaction with ZO-1. We further hypothesize that overexpression of truncated Bves in epithelial cells results in disruption of tight junctions, increased RhoA activation by free GEF-H1, and increased ZONAB/Dbpa transcriptional activity in the nucleus.

TJs have been traditionally viewed as mechanical protein structures functioning in barrier formation, regulating paracellular flow of fluids and small solutes [11], [12]. However, TJs have been shown by Balda and Matter to play a direct role in regulation of Rho and in gene transcriptional regulation [13], [14], [15]. GEF-H1 is an activator of RhoA sequestered within TJs (Figure 2). ZONAB/DbpA, a y-box transcription factor, is also associated with TJs through direct interaction with ZO-1. ZONAB/DbpA binds directly with ZO-1, while GEF-H1 complexes indirectly with ZO-1 through the adapter protein cingulin (Figure 2). Interestingly, we have demonstrated by GST pull-down that the intracellular carboxyl terminus of Bves interacts with ZO-1 [1]. We postulate that Bves regulates RhoA activation and ZONAB/DbpA transcriptional activity through modulation of TJ formation.

To test our hypothesis, human corneal epithelial cells (HCE) are stably transfected to overexpress either a wild-type Bves or a mutant Bves truncated within the conserved intracellular C-terminus Popeye domain (Figure 1). The over-expression of the truncated mutant Bves (t-Bves) appears to disrupt the function of endogenous Bves by interfering with cell membrane trafficking (Figure 2). Failure of Bves to localize to sites of cell-cell contact in t-Bves cells is associated with decreased TJ formation as indicated by reduced localization of TJ proteins at the cell membrane and lowered trans-epithelial electrical resistance. We further show that t-Bves cells exhibit increased RhoA and ZONAB/Dbpa activation, which are due to decreased sequestration of GEF-H1 and ZONAB/Dbpa at the cell membrane. These findings indicate that Bves coordinates both TJ associated RhoA and ZONAB/Dbpa activation.

## Results

### Stable overexpression of t-Bves disrupts localization of endogenous Bves at cell-cell junctions

**Figure 3:**
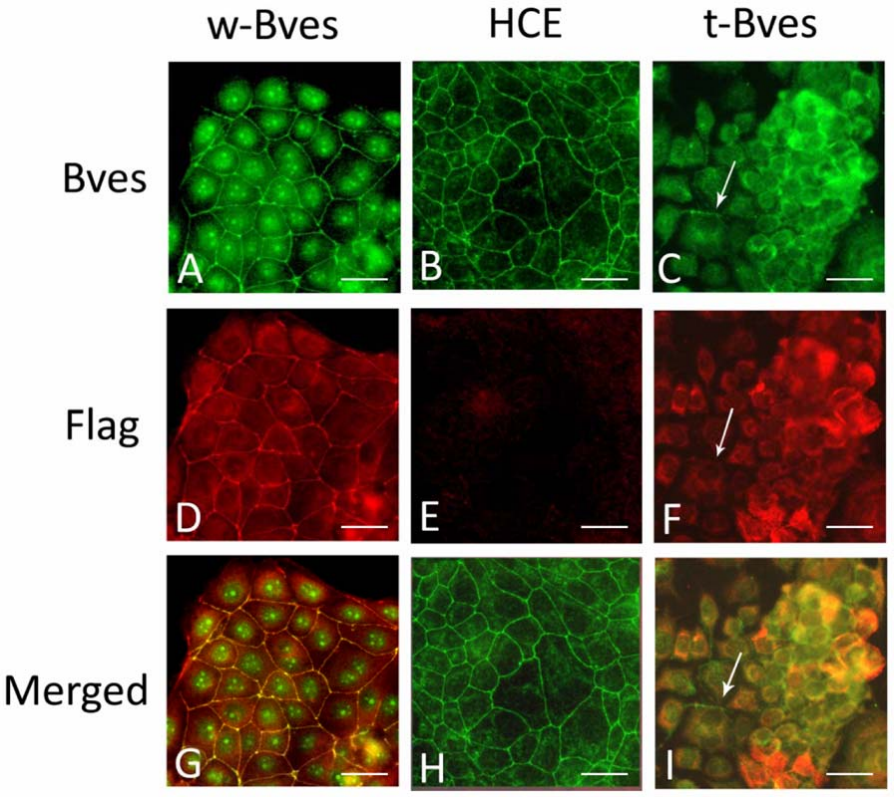
Immunostaining of endogenous Bves and Flag-tagged constructs. Flag-tagged chicken Bves constructs were localized to cell borders in w-Bves cells (D) in a pattern similar to endogenous Bves in wild type cells (B). As the t-Bves construct does not contain the anti-Bves antibody binding site, Bves immunostaining seen in t-Bves cells (C) was only endogenous Bves. Endogenous Bves co-localized with Flag-tagged t-Bves in the cytoplasm of cells (I, yellow). Some endogenous Bves (green) was seen at cell borders without co-localized t-Bves (I, arrow). (Scale bar 50 µm).

Bves is an integral epithelial TJ protein, and its function is dependent on proper subcellular trafficking to the cell membrane, which in turn is dependent on homotypic interactions between Bves molecules [8], [9]. When human corneal epithelial (HCE) cells were stably transfected with full length Flag-tagged chicken Bves (w-Bves) immunostaining for Flag was localized to the cell membrane in a wire-mesh pattern (Figure 3D) similar to immunostaining for endogenous Bves in the parental HCE cells (Figure 3B). In cells overexpressing C-terminus truncated Bves (t-Bves), we did not observe Flag immunostaining of t-Bves at cell-cell borders (Figure 3F). Our antibody to Bves binds a region of the cytoplasmic tail that was deleted from the t-Bves construct (Figure 1). Therefore, Bves immunostaining of t-Bves cells (Figure 3C) shows localization of endogenous Bves only. There were occasional areas of only endogenous Bves localizing at the cell border (Figure 3C, arrow). However, we primarily observed an accumulation of both endogenous Bves (Figure 3C) and t-Bves (Figure 3F) in the cytoplasm. This overlapping t-Bves and endogenous Bves immunolocalization (Figure 3I, yellow) suggested that stably transfected t-Bves was interacting with endogenous human Bves. However, interaction with t-Bves appeared to disrupt the cell membrane localization of the endogenous Bves.

**Figure 4:**
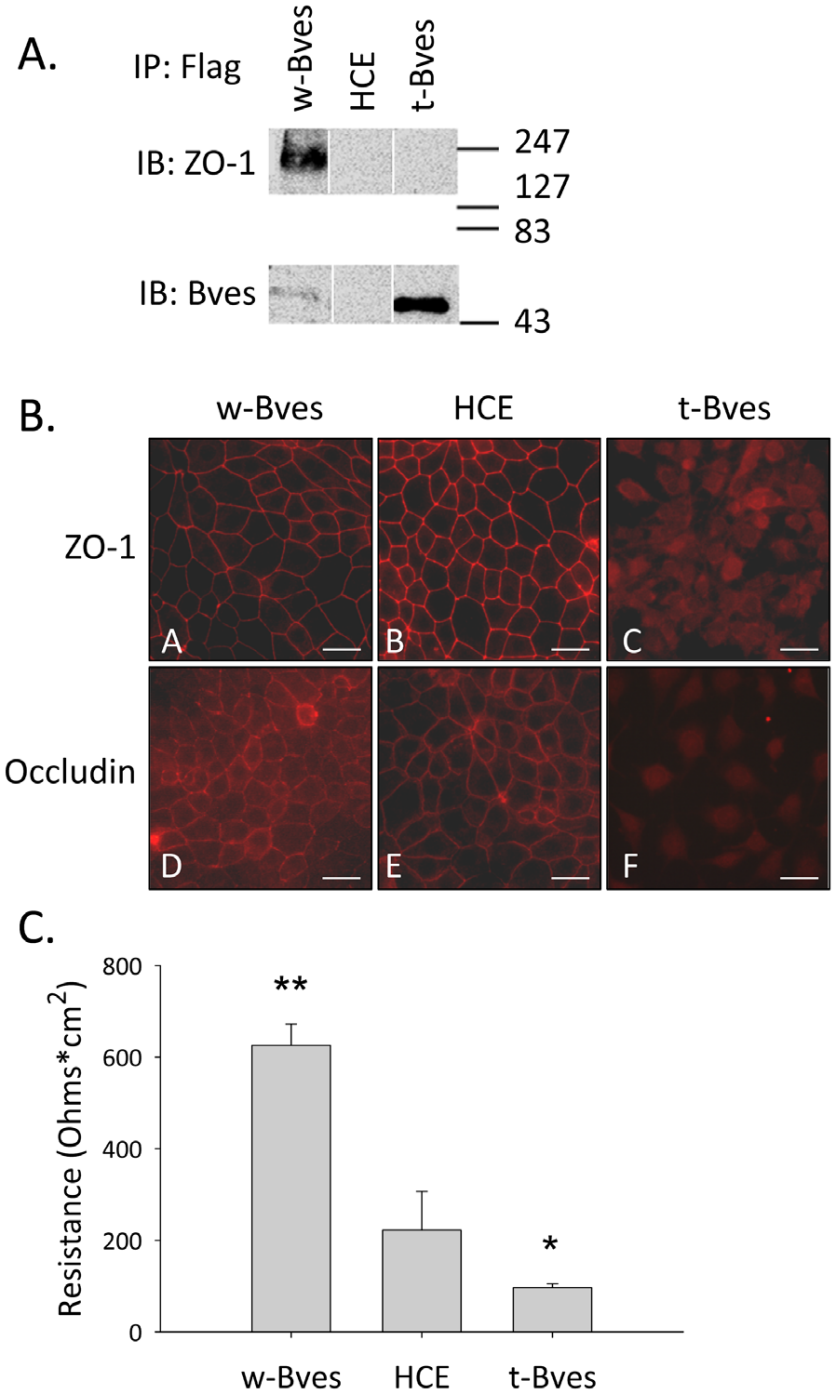
Flag immunoprecipitation, TJ protein immunostaining and Trans-Epithelial Electrical Resistance. A. Whole cell lysates from w-Bves, parental HCE, and t-Bves cells were precipitated with Flag antibody and analyzed by Western blot for endogenous Bves and ZO-1. The lanes shown were run as a single gel, but extraneous lanes have been omitted and replaced with white lines. As expected, Bves was pulled down from w-Bves cells but not parental HCE cells. Endogenous Bves was also pulled down with truncated Flag-tagged t-Bves. ZO-1 was also pulled down with the Flag-tagged construct from the w-Bves cells, but Bves was not pulled down from parental HCE cells nor was ZO-1 pulled down with the truncated Flag-tagged construct from the t-Bves cells. B. TJ proteins ZO-1 and occludin were both localized to cell borders in w-Bves (A, D) and HCE cells (B, E), but immunostaining for these proteins was diffuse in t-Bves cells (C, F). C. Trans-epithelial electrical resistance (TER), an indicator of TJ function, was significantly increased in w-Bves cells and compared to HCE (P<0.005), while TER was significantly reduced in t-Bves cells (p<0.05).

Immunoprecipitation studies were carried out to verify that the stably expressed Flag-tagged constructs, w-Bves and t-Bves, interact with endogenous human Bves. Whole cell lysates from w-Bves, parental HCE, and t-Bves cells were precipitated with Flag antibody and analyzed by Western blot for endogenous Bves and ZO-1 (Figure 4A). As expected, the Flag antibody precipitated Bves in w-Bves cells overexpressing full-length Flag-tagged Bves. The untransfected parental HCE cells did not precipitate Bves, as there was no Flag present to pull down. However, endogenous Bves was precipitated by Flag antibodies in t-Bves cells. As our antibody to Bves (846) does not bind to the truncated t-Bves construct, this indicates an interaction between the endogenous Bves seen in the blot and the Flag-tagged truncated Bves.

We have shown by GST pull-down with MDCK cell lysates that ZO-1 interacts with a C-terminal domain of Bves [1]. In this study, we found that ZO-1 was also precipitated by Flag, indicating an interaction between w-Bves and ZO-1 within w-Bves cells. This is the first demonstration of a Bves-ZO-1 interaction within whole cells. As expected, untransfected parental HCE cells did not precipitate ZO-1. Interestingly, the interaction between endogenous Bves and t-Bves appeared to disrupt the interaction with ZO-1 in t-Bves cells, as ZO-1 was not precipitated by Flag.

The interaction between w-Bves and endogenous Bves appeared to retain normal Bves cell membrane localization (Figure 3G) and interaction with ZO-1 (Figure 4A). In contrast, interaction between t-Bves and endogenous Bves disrupted Bves trafficking to the cell membrane (Figure 3I) and had attenuated capacity to interact with ZO-1 (Figure 4A). Thus, t-Bves may exert a negative functional effect on endogenous Bves by disrupting cell membrane localization and interactions with ZO-1.

### Decreased Tight Junctions in t-Bves cells

Since ZO-1 is a critical component of TJs, immunolocalization studies were carried out to evaluate the effect of t-Bves on TJ protein cell localization. ZO-1 was seen at the cell membrane in w-Bves and HCE cells (Figure 4B A, B). In contrast, ZO-1 was only seen in a cytoplasmic distribution (Figure 4B C), even at sites of apparent cell-cell contact. The inability of t-Bves cells to form cell-cell adhesions was further supported by displacement of occludin from the cell membrane in t-Bves cells (Figure 4B F). Occludin was readily seen at the cell membrane in w-Bves and parental HCE cells in a wire-mesh pattern (Figure 4B D, E).

Transepithelial electrical resistance (TER) was measured in order to determine the effect of t-Bves expression on TJ function. TER was approximately doubled in w-Bves cells compared to HCE cells (Figure 4C), which is consistent with our previous report [1]. In contrast, t-Bves cells exhibited a decrease in TER compared to HCE cells (Figure 4C). Together, these findings indicate that expression of t-Bves resulted in the failure of endogenous Bves to localize to the cell membrane which disrupted the establishment of cell-cell adhesions and the formation of functioning TJs.

### Overexpression of t-Bves results in increased RhoA and ZONAB/DbpA activity

**Figure 5:**
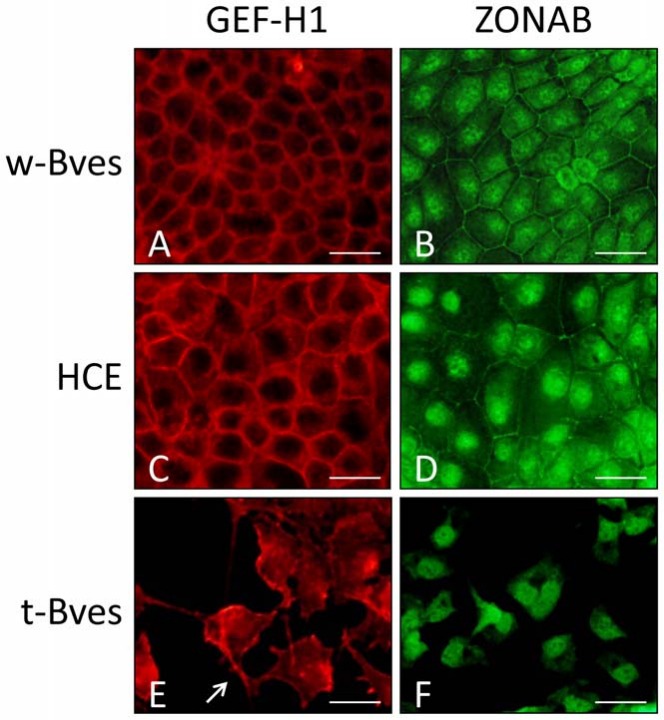
GEF-H1 and ZONAB/Dbpa immunostaining. GEF-H1 (A) and ZONAB (B) were found at cell-cell borders in monolayers of w-Bves cells. A similar staining pattern was seen in parental HCE cells (C and D). In t-Bves cells, GEF-H1 was seen in podial extensions of migrating cells (E arrow), and ZONAB staining was seen primarily in the nucleus (F). (Scale bar 50 µm).

The disruption of TJ formation by t-Bves expression in HCE cells led to the hypothesis that Bves plays a role in cell signaling through the sequestration of GEF-H1, an activator of RhoA, and ZONAB/DbpA, a y-box transcription factor (Figure 2). Immunolocalization studies were performed to evaluate changes in GEF-H1 and ZONAB/DbpA localization associated with Bves expression and localization (Figure 5). In w-Bves and parental HCE cells GEF-H1 was seen at the cell border (Figure 5A, C) in the same pattern as ZO-1 (Figure 4A, B). However, expression of t-Bves led to increased cytoplasmic localization of GEF-H1 (Figure 5E). GEF-H1 was also seen in podial extensions (Figure 5E arrow) similar to the findings of Tsapara et al in TGF-β stimulated retinal pigment epithelial cells [16]. ZONAB/Dbpa immunostaining was seen at cell borders in w-Bves and HCE cells (Figure 5B, D) but was primarily found in the nucleus of t-Bves cells (Figure 5F) without cell membrane localization even at sites of cell-cell contact. We found that ZONAB/DbpA, GEF-H1, and ZO-1 localize to cell-cell junctions in w-Bves and parental HCE cells as expected. However, expression of t-Bves appeared to disrupt the TJ complex resulting in increased GEF-H1 and ZONAB/DbpA away from cell-cell junctions.

**Figure 6:**
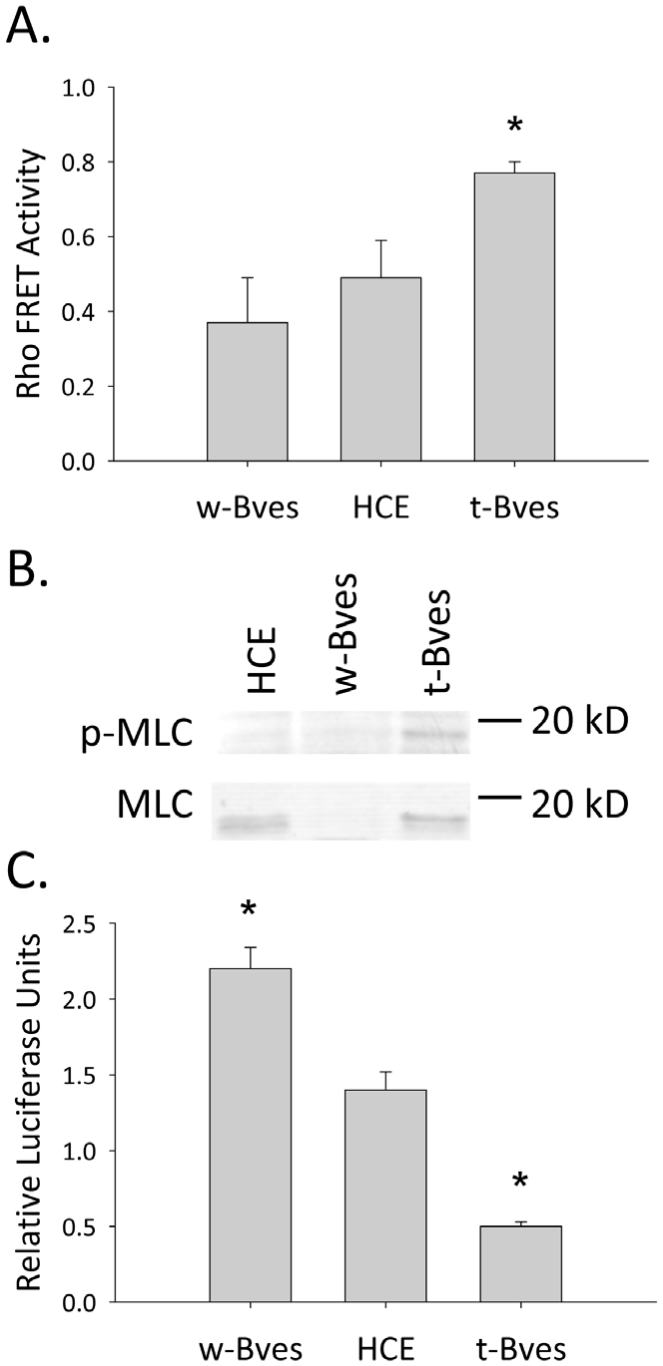
RhoA and ZONAB/Dbpa activity. A. Rho activity was assessed using Raichu FRET probes. RhoA activity was slightly decreased in w-Bves cells compared to parental HCE cells. In t-Bves cells, RhoA activity was significantly increased compared to parental HCE cells (p<0.05) B. Western blot dual-stained for phosphorylated myosin light chain (p-MLC) and total myosin light chain (MLC) is shown. MLC is a downstream target of active RhoA. The ratio of p-MLC to total MLC was not significantly different between w-Bves cells and parental HCE cells. However, the ratio of p-MLC to total MLC in t-Bves cells was 20 times greater than in parental HCE cells (p<0.005), supporting our observed increase in RhoA activity. C. ZONAB/Dbpa activity was monitored using a luciferase reporter assay. Decreased Relative Luciferase Units indicates increased ZONAB activity. Therefore, ZONAB activity was significantly reduced in w-Bves cells (p<0.05) and increased in t-Bves cells (p<0.05) compared to parental HCE cells.

We suspected that the increase in free GEF-H1, an activator of RhoA, would be associated with increased RhoA activation (Figure 2). The state of RhoA activation was assessed using a RhoA-FRET probe, where increased FRET activity is associated with increased levels of activated RhoA. w-Bves cells exhibited a trend towards decreased FRET activity compared to HCE cells which was not statistically significant. However, in t-Bves cells we observed a significant 30% increase in FRET activity compared to parental HCE cells (Figure 6B).

Next, we examined the phosphorylation of myosin light chain (MLC), a downstream target of RhoA. The ratio of phosphorylated MLC (p-MLC) to total MLC was slightly decreased in w-Bves cells compared to HCE cells (Figure 6C). In contrast, the ratio of p-MLC to total MLC was 20 times greater in t-Bves cells compared to HCE cells (Figure 6C, N = 3, p<0.05), confirming the increase in RhoA activity reported by the FRET assay.

Similarly, we expected to find increased ZONAB/DbpA activity in t-Bves cells. The state of ZONAB/DbpA transcriptional activity was assessed using a luciferase reporter probe. In this assay, a decrease in the ratio of fire fly luciferase over renilla luciferase (ff/r) indicates increased ZONAB/DbpA transcriptional activity. We found that w-Bves cells had a significantly higher ff/r luciferase ratio compared to HCE cells (Figure 6D), reflecting decreased ZONAB/DbpA transcriptional activity in cells with increased Bves expression. Conversely, t-Bves cells exhibited significantly lower ff/r luciferase ratio compared to HCE cells (Figure 6D) which indicates increased ZONAB/DbpA activity. These findings support our hypothesis that Bves expression and localization can inversely regulate RhoA and ZONAB/DbpA activity through regulation of the TJ complex.

## Discussion

Bves is an adhesion molecule expressed in a variety of tissues [17], [18], [19]. We previously reported that epithelial TJ formation is directly related to Bves levels [1], [9]. In addition, we observed that reduction of Bves expression altered the epithelial phenotype, leading to increased cellular motility [9], [18]. These effects on cellular function implicate Bves in a role beyond the mechanical roles of TJs in cell adhesion and epithelial barrier formation [5]. This is the first report of an interaction between Bves and ZO-1 in whole cells (Figure 4A). When the interaction between ZO-1 and Bves is inhibited, ZO-1 does not localize to the cell membrane (Figure 4B), tight junction barrier function is reduced (Figure 4C), and tight junction associated signaling through RhoA and ZONAB/DbpA is altered (Figure 6). Therefore, we hypothesize that Bves plays a role in cell-cell contact inhibition by regulating formation of TJs which coordinate RhoA and ZONAB/DbpA transcriptional activities that result in decreased motility and cell cycling [13], [16]. These findings provide new insights into the role of TJs as cell signaling structures and novel pathways regulating TJ formation.

### The carboxyl domain is critical for Bves localization to the cell membrane

This study provides functional evidence that alteration of the highly conserved Popeye domain can lead to altered cell functions. We have previously shown that decreased Bves protein is associated with decreased TJs [1]. The current study verifies that cell membrane localization is critical for Bves' role in regulating TJ formation (Figure 4). Findings reported in this study also indicate that the trafficking of Bves to the cell membrane requires homotypic interactions between Bves molecules. This is the first study to provide direct in vivo evidence for the occurrence of Bves-Bves interactions. In our immunoprecipitation studies using Flag antibodies to precipitate stably transfected w-Bves and t-Bves, we observe endogenous Bves interacting with both full length w-Bves and truncated t-Bves (Figure 4). w-Bves/endogenous Bves heterogeneous complexes are seen at the cell membrane in the same localization pattern as endogenous Bves (Figure 3). In contrast, t-Bves/endogenous Bves heterogeneous complexes are only seen in the cytoplasm (Figure 3). The interaction between t-Bves and endogenous Bves results in an incomplete pairing of the intracellular carboxyl terminal tail (Figures 1 and 2). This incomplete pairing of the carboxyl terminus leads to disrupted cell membrane localization and increased endogenous Bves in the cytoplasm.

We have reported an intracellular Bves-Bves interaction domain within the carboxyl terminus (aa 268–274), with 2 conserved lysine residues (K^272^ and K^273^) [9]. Transfection of HCE cells with Bves constructs having K^272^ and K^273^ deleted (KKD-Bves) or mutated to alanines (KKM-Bves) led to decreased TJ formation along with cytoplasmic accumulation of endogenous Bves. However, unlike t-Bves cells, after two weeks KKD-Bves and KKM-Bves cells regained the ability to organize into epithelial sheets, and KKD-Bves and KKM-Bves were seen at the cell membrane (Figure S1). Furthermore, other critical TJ proteins were found at the cell membrane. These findings suggest that these mutations of the intracellular interaction domain (aa 268–274) do not prevent formation of TJ cell adhesions as seen with the t-Bves mutation. It is possible that the role of Bves' intracellular interaction domain is in the initial alignment of Bves molecules, leading to efficient formation and trafficking of Bves to the cell membrane.

### The carboxyl domain is critical for Bves interaction with ZO-1

Prior evidence suggesting Bves/ZO-1 interaction in cells was indirect, based on immunolocalization studies demonstrating close proximity of ZO-1 and Bves at the cell membrane and on in vitro GST pull down assays with a carboxyl terminal Bves-GST construct to pull down ZO-1 from whole cells lysates [1], [18]. This is the first report demonstrating Bves/ZO-1 interactions in vivo, and it appears the pairing of Bves' carboxyl domain is needed for interaction with ZO-1 (Figure 4).

The biological relevance of Bves/ZO-1 interaction is not fully known, but we suspect that this interaction is critical for Bves' regulatory effect on TJ formation. ZO-1 is considered to be a scaffolding protein, linking TJ transmembrane proteins to cytoplasmic proteins and cytoskeletal filaments. However, there is growing evidence that ZO-1 plays an active role regulating TJ assembly. In studies either disrupting ZO-1 expression, or expressing a mutant ZO-1, TJ assembly was disrupted [20], [21], [22]. Given that our study demonstrates Bves localization to be critical for TJ formation, and that Bves/ZO-1 interaction occurs in vivo, it is not unreasonable to suspect that Bves' regulatory effect on TJs is through its interaction with ZO-1.

### Bves Coordinates Cellular Signaling

The findings in this study also demonstrate that Bves can modulate TJ associated signaling pathways RhoA and ZONAB/DbpA. Cells overexpressing t-Bves exhibit significantly higher levels of RhoA activity and ZONAB/DbpA activity compared to parental HCE cells (Figure 6). Conversely, in cells with increased Bves expression (w-Bves) there is significantly lower ZONAB/DbpA activity. While there was only a trend towards decreased active RhoA in the w-Bves cells, we have recently reported that overexpression of Bves significantly reduces RhoA activation in trabecular meshwork cells [10]. Thus, we suspect that the trend towards decreased active RhoA is a true biologic effect of increased Bves in the HCE cells. These changes in active RhoA and ZONAB/DbpA activity appear to be correlated with GEF-H1 and ZONAB/DbpA cellular localization. w-Bves and parental HCE cells exhibit cell membrane localization of both GEF-H1 and ZONAB/DbpA. In contrast, t-Bves cells exhibit cytoplasmic and nuclear localization of GEF-H1 and ZONAB/DbpA, respectively, which correlates with increased active RhoA and ZONAB/DbpA activity. These observations indicate that Bves modulates TJ associated RhoA signaling and ZONAB/DbpA transcriptional activity.

Interestingly, Smith et al recently reported that Bves can directly modulate the activity of another Rho GTPase activator, GEFT [23]. Smith et al. demonstrated by using yeast two-hybrid screening that the carboxyl region of Bves binds directly with GEFT. In addition, overexpression of the carboxyl region without the transmembrane portion of Bves leads to decreased Rac and Cdc42 activation, but not RhoA. From this report, it is unclear how Bves interaction with GEFT regulates GEFT activity. Based on these observations and our current findings, it is tempting to speculate that through Bves there is convergence or coordination of multiple signaling pathways. Furthermore, these pathways are potentially the molecular basis for the role of Bves in governing epithelial cell motility, adhesion and differentiation, which are critical for organogenesis and wound healing [17], [24], [25].

## Materials and Methods

### Development of w-Bves and t-Bves HCE lines

Immortalized human corneal epithelial cells [26] were stably transfected using Lipofectin 2000 according to the manufacturer's instructions (Invitrogen) and plasmid. Full-length chick Bves with Flag tag [1] was used to over-express wild type Bves (w-Bves). We used a plasmid coding the Flag-tagged chick Bves sequence truncated at aa214 (within the Popeye domain) to create a dominant negative cell line with disrupted Bves (t-Bves). Transfected cells were selected using medium containing G418 (Cellgro; 30 µg/ml). Resistant cells were maintained in medium containing 20 µg/ml G418.

### Antibodies

Polyclonal antibodies against ZO-1 and occludin were purchased from Zymed-Invitrogen. The polyclonal antibody against Bves (846) was obtained from Dr. David Bader's laboratory (Vanderbilt University Medical Center, Nashville, TN) [24]. Monoclonal anti-GEF-H1 antibody was the gift of Dr. Karl Matter (Institute of Ophthalmology, University College, London). Rabbit anti-ZONAB polyclonal antibody was generated by Enzyme Antibodies (San Francisco, CA) against aa 174–187 of the canine ZONAB-A sequence. Monoclonal anti-phospho-myosin light chain 2 and polyclonal anti-myosin light chain 2 antibodies were purchased from Cell Signaling Technology, Inc (Danvers, MA). Flag antibodies were purchased from Sigma (St Louis, MO). Alexa Fluor 488 and Alexa Fluor 568 secondary antibodies were purchased from Molecular Probes-Invitrogen. For western blot, IRDye 800 secondaries were purchased from Rockland Immunochemicals (Gilbert, PA), and Alex Flour 680 goat anti-rabbit was from Molecular Probes-Invitrogen.

### Western blotting

Protein extraction and western blotting were performed as previously described [1], [9], [18]. For phosphorylated myosin light chain, the same blot was dual stained for phosphorylated myosin light chain and total myosin light chain. AlexaFluor 680 or IRDye 800 conjugated secondary antibodies were applied, and blots were scanned and analyzed (Odyssey Infrared Imaging System; Li-Cor Biosciences).

Densitometric measurements were made using Image Pro Plus. Each area of interest was background subtracted. Phosphorylated myosin light chain was divided by total myosin light chain. To account for exposure differences between blots, all samples were normalized to the parental HCE samples. Normalized density values were compared using a one-way analysis of variance among all groups followed by an all pair-wise multiple comparison (p<0.05).

### Immunoprecipitation

The Pierce Seize X Protein G Immunoprecipitation Kit (45210) was used according to manufacturer's instructions. Confluent cultured cells were washed, physically dissociated from culture dish, and lysed in provided kit lysis buffer. BCA protein assay kit (Pierce) was used to determine protein concentration of lysate. Total protein of each sample was adjusted to 400 µg before addition of primary antibody. Kit instructions were followed for immobilization of secondary antibody to protein G beads and immunoprecipitation. Western blotting of immunoprecipitated samples was performed as described above.

### Immunofluorescence

Immunofluorescence staining was carried out using cultured HCE cells fixed in 70% methanol for 15 min., permeabilized in PBS with 0.25% Triton X-100 for 10 min., and blocked with PBS containing 2% BSA for 1 hour at room temperature. Primary antibodies were diluted in 1% BSA and incubated overnight at 4°C. The cells were washed with PBS, and secondary antibodies were added for 3 hours at room temperature. After PBS and water washes, images were mounted in Aquapoly mounting media and captured using a Nikon fluorescence microscope.

### Transepithelial electrical resistance

Electrical resistance was measured using the method of Oshima et al. [27]. Briefly, HCE cells were seeded at 5,000 cells/cm^2^ in six-well polycarbonate tissue culture inserts with 0.4 µm pores (BD Falcon, San Jose, CA). Cells were cultured for 14 days. Prior to measurement, inserts were washed with 37°C PBS with calcium and magnesium. Two ml of PBS was added to the chamber containing the lower electrode, the insert was placed in the chamber, 2 ml of PBS was added to the insert, and the upper electrode was positioned in the PBS in the insert. The resistance of the control insert was subtracted from the cell measurements. The background corrected measurement was multiplied by the area of the filter. These resistance values were compared using a one-way analysis of variance among all groups followed by an all pair-wise multiple comparison (p<0.05).

### RhoA activation detected by FRET probes

We evaluated the state of RhoA activation using FRET probes (fluorescence resonance energy transfer). RhoA-Raichu FRET plasmids (gift from Dr. Michiyuki Matsuda, Osaka, Japan) were transfected into HCE cells at 50% confluence. In addition, some HCE cells also received empty vector, Flag-tagged full length chicken Bves, or truncated chicken Bves. Thirty-six hours later, whole lysates from the transfected cells were evaluated for FRET according to the method of Aijaz et al. [14]. Briefly, a Perkin Elmer LS50B fluorescence spectrophotometer was configured for 430 nm (CFP, FRET) and 517 nm (YFP) excitations and 475 nm and 530 nm emissions. Measurements were taken at (excitation/emission wavelength) 430/475 (CFP), 430/530 (FRET), and 517/530 (YFP). Non-transfected cells and cells transfected with CFP or YFP constructs only were used as background and single-channel cross-talk controls. Net FRET signal, including compensation for background and cross-talk, was calculated according to the method of Chusainow et al. [28]. Net FRET efficiency values for the cell lines were compared by unpaired t-test (p<0.05).

### ZONAB/DbpA activity detected by Luciferase reporter assay

A dual luciferase reporter assay used to quantify ZONAB/DbpA activity was a gift from Dr. Karl Matter, University College London, and was used as previously reported [29]. In this assay, an ErbB2 promoter containing a ZONAB/DbpA-binding site is utilized to regulate firefly luciferase expression. As a control, a mutated promoter of similar length which is incapable of binding ZONAB/DbpA was used to regulate Renilla luciferase expression. Both plasmids were co-transfected into HCE cells using FuGENE 6 (Roche) according to manufacturer instructions. Forty eight hours post-transfection, cell lysates were measured ratiometrically for dual luciferase activity using the Promega dual luciferase reporter assay kit according to manufacturer instructions, on a dual-injection Lumimark Plus microplate luminometer (Bio-Rad Inc.). Results were compared using one-way ANOVA (Sigmaplot 11, Systat) with SNK post test. p<0.05 was considered significant.

## Supporting Information

Figure S1 Immunostaining for Bves, Flag, E-cadherin, occludin, and ZO-1. Membrane localization of mutant Bves constructs and formation of TJs in KKD-Bves and KKM-Bves is delayed in KKD-Bves and KKM-Bves cells [9]. However, after two weeks in culture, endogenous Bves and mutant Bves constructs are seen at cell borders (A and B, scale bar 20 um). E-cadherin and the TJ proteins occluding and ZO-1 are also found at cell borders (C-H, scale bar 50 µm).
(2.91 MB TIF)
