# Supplementary figures and images for: Bves Modulates Tight Junction Associated Signaling
Source: PLoS One. 2011 Jan 20;6(1):e14563. doi: 10.1371/journal.pone.0014563 (PMC3024319; doi:10.1371/journal.pone.0014563)

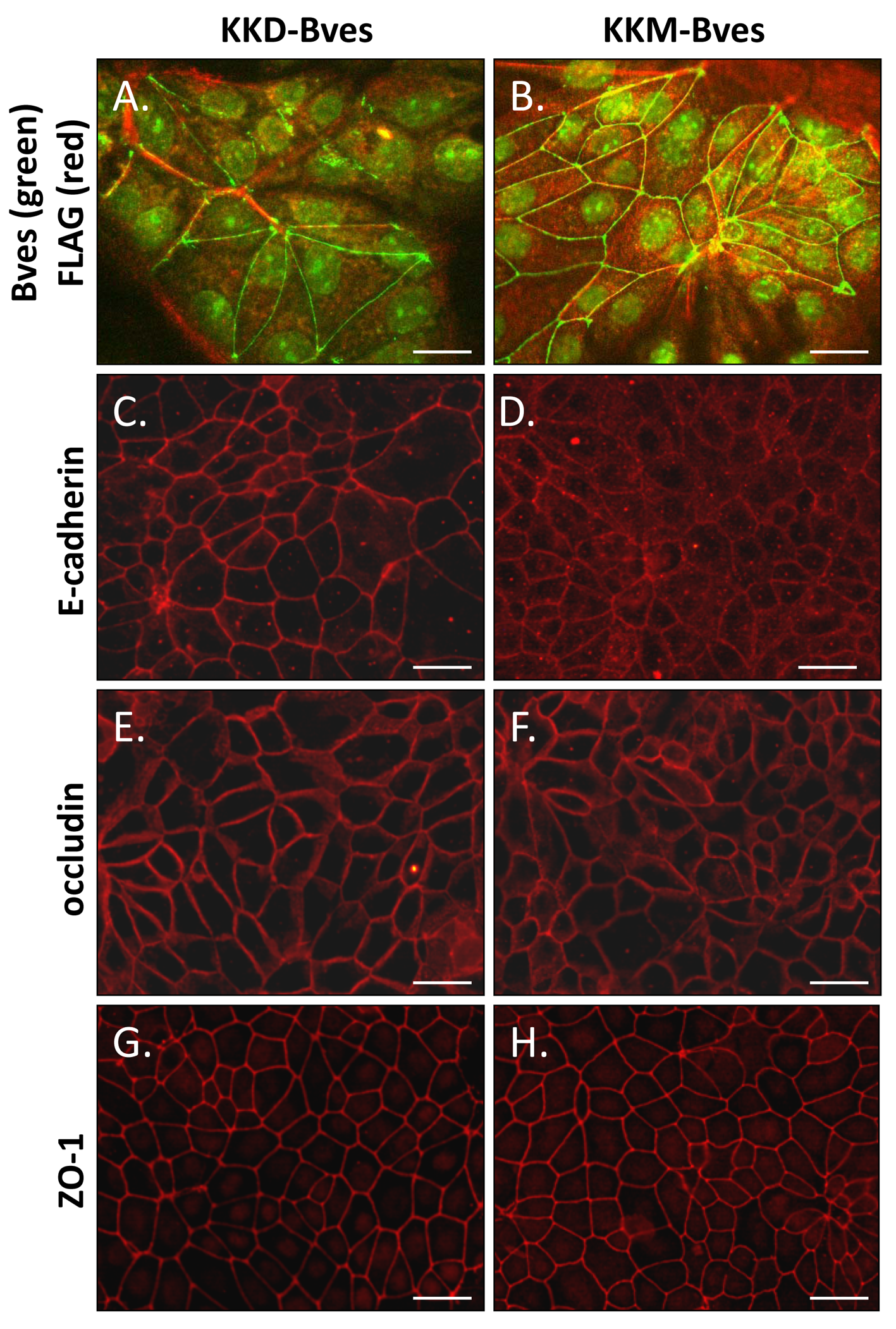

Supplement: Figure S1 — Immunostaining for Bves, Flag, E-cadherin, occludin, and ZO-1. Membrane localization of mutant Bves constructs and formation of TJs in KKD-Bves and KKM-Bves is delayed in KKD-Bves and KKM-Bves cells [9]. However, after two weeks in culture, endogenous Bves and mutant Bves constructs are seen at cell borders (A and B, scale bar 20 um). E-cadherin and the TJ proteins occluding and ZO-1 are also found at cell borders (C-H, scale bar 50 µm). (2.91 MB TIF) [file pone.0014563.s001.tif]
